# University of California

**Published:** 1895-11

**Authors:** 


### AMONG THE COLLEGES.

### UNIVERSITY OF CALIFORNIA.

THE veterinary department opened its new year on October 1st. As before announced, it will be a curriculum of three years of six months each.

Dr. F. A. Nief, formerly dean of the school, has been succeeded by Dr. F. W. Skaife. The faculty will have five well-known veterinarians on the Pacific Coast among the corps of instructors. The course will be graded over the three years, embracing the first year chemistry and materia medica, elements of physiology and histology, anatomy, dissection, and botany; second year: physiology and comparative physiology, anatomy of the domesticated animals and comparative anatomy, principles and practice of veterinary surgery, principles and practice of equine medicine, principles and practice of bovine medicine, materia medica, analytical and organic chemistry, toxicology, dissection, clinical instruction, and therapeutics; third year: principles and practice of veterinary surgery, principles and practice of equine medicine and dermatology, principles and practice of bovine medicine, principles and practice of canine medicine and helminthology, veterinary obstetrics, bacteriology, pathological anatomy, veterinary hygiene, clinical instruction, and therapeutics—a plan which we hope will be ultimately adopted by all the veterinary schools of North America. The students, beside the daily clinical instruction, will be assigned places as hospital aids in the second year, and cases in the hospital the third. The degree of Doctor of Veterinary Science (D.V.S.) will be conferred upon the graduates.

The matriculation examination will be written and will correspond to a high-school requirement for graduation, allowances being made to agricultural college graduates, also those of college of pharmacy and medical and other veterinary colleges. Great care and explicit rules are laid down under the head of matriculation. The requirements for graduation are thorough, and the school starts out on a well-defined course of advanced higher veterinary education.

